# A cluster randomized controlled trial for the Evaluation of routinely Measured PATient reported outcomes in HemodialYsis care (EMPATHY): a study protocol

**DOI:** 10.1186/s12913-020-05557-z

**Published:** 2020-08-10

**Authors:** Jeffrey A. Johnson, Fatima Al Sayah, Robert Buzinski, Bonnie Corradetti, Sara N. Davison, Meghan J. Elliott, Scott Klarenbach, Braden Manns, Kara Schick-Makaroff, Hilary Short, Chandra Thomas, Michael Walsh

**Affiliations:** 1 2-040 Li Ka Shing Centre for Health Research Innovation, School of Public Health, University of Alberta, Edmonton, AB T6G 2E1 Canada; 2 Patient Partner, Medicine Hat, Alberta Canada; 3 11-113L Clinical Sciences Building, Division of Nephrology and Immunology, Department of Medicine, University of Alberta, Edmonton, AB T6G 2G3 Canada; 4 Foothills Medical Centre, 1403 - 29th Street NW, Calgary, AB T2N 2T9 Canada; 5 11-107 Clinical Sciences Building, Division of Nephrology and Immunology, Department of Medicine, University of Alberta, Edmonton, AB T6G 2G3 Canada; 6 Department of Medicine and Department of Community Health Sciences, University of Calgary, Calgary, Canada; 7 5-295 Edmonton Clinic Health Academy, Faculty of Nursing, University of Alberta, Edmonton, AB T6G 1C9 Canada; 8 Division of Nephrology, Department of Medicine, McMaster University, Marion Wing, Level 3, St. Joseph’s Healthcare, 50 Charlton Ave. E, Hamilton, ON L8N 4A6 Canada

**Keywords:** Kidney failure, Hemodialysis, Patient-reported outcome measures, Symptom burden, Quality improvement, Controlled trial

## Abstract

**Background:**

Kidney failure requiring dialysis is associated with poor health outcomes and health-related quality of life (HRQL). Patient-reported outcome measures (PROMs) capture symptom burden, level of functioning and other outcomes from a patient perspective, and can support clinicians to monitor disease progression, address symptoms, and facilitate patient-centered care. While evidence suggests the use of PROMs in clinical practice can lead to improved patient experience in some settings, the impact on patients’ health outcomes and experiences is not fully understood, and their cost-effectiveness in clinical settings is unknown. This study aims to fill these gaps by evaluating the effectiveness and cost-effectiveness of routinely measuring PROMs on patient-reported experience, clinical outcomes, HRQL, and healthcare utilization.

**Methods:**

The EMPATHY trial is a pragmatic multi-centre cluster randomized controlled trial that will implement and evaluate the use of disease-specific and generic PROMs in three kidney care programs in Canada. In-centre hemodialysis units will be randomized into four groups, whereby patients: 1) complete a disease-specific PROM; 2) complete a generic PROM; 3) complete both types of PROMs; 4) receive usual care and do not complete any PROMs. While clinical care pathways are available to all hemodialysis units in the study, for the three active intervention groups, the results of the PROMs will be linked to treatment aids for clinicians and patients. The primary outcome of this study is patient-provider communication, assessed by the Communication Assessment Tool (CAT). Secondary outcomes include patient management and symptoms, use of healthcare services, and the costs of implementing this intervention will also be estimated. The present protocol fulfilled the Standard Protocol Items: Recommendations for Intervention Trials (SPIRIT) checklist.

**Discussion:**

While using PROMs in clinical practice is supported by theory and rationale, and may engage patients and enhance their role in decisions regarding their care and outcomes, the best approach of their use is still uncertain. It is important to rigorously evaluate such interventions and investments to ensure they provide value for patients and health systems.

**Trial registration:**

Protocol version (1.0) and trial registration data are available on www.clinicaltrials.gov, identifier: NCT03535922, registered May 24, 2018.

## Background

At the end of 2018 there were over 40,000 Canadians with kidney failure requiring dialysis; a 35% increase from the previous decade [1]. Kidney failure requiring dialysis is associated with high symptom burden and poor health-related quality of life (HRQL) [2] [3] [4] [5]. Patients can experience a wide variety of symptoms ranging from physical to psychological in nature [6]. These symptoms are often under-recognized by kidney care teams, which may be related to breakdown in communication [7]. In a recent priority setting exercise, the top research priority for patients with kidney failure requiring dialysis in Canada was to enhance communication between patients and clinicians to maximize patient participation in decision making and facilitate self-management [8]. Furthermore, five of the top 10 research priorities for patients with kidney failure requiring dialysis focus on reducing symptoms and improving HRQL [6].

The priorities of enhancing communication and improving symptoms and HRQL are potentially well aligned. In other chronic diseases, the incorporation of routinely measuring and reporting symptoms and HRQL improved communication [9–11]. Without routine symptom monitoring, this situation is unlikely to change. Further, the presence and severity of symptoms can change considerably over a short period of time in dialysis patients, emphasizing the need for regular surveillance of symptoms. Incorporation of patient-reported outcome measures (PROMs) into dialysis care could address this problem.

### What are PROMs?

PROMs are reports coming directly from patients about how they function or feel in relation to a health condition and its therapy, without interpretation of the patient’s responses by a clinician or anyone else [12]. PROMs present an opportunity to explore aspects of patients’ health that may otherwise be overlooked. They capture patients’ experiences of symptoms and impact of disease on functioning and can support clinicians to monitor disease progression and facilitate patient-centered care. It is proposed that PROMs could act to improve the quality of care in the same way as any other benchmarking tool [13], and some suggest that PROMs have the potential to transform healthcare [14]. There has been an increased use of PROMs in health systems around the world, a movement initiated and led by the National Health System (NHS) in the UK [13]. In Canada, the Canadian Institute for Health Information (CIHI) and the Alberta PROMs and EQ-5D Research and Support Unit [15] are leading similar efforts to establish provincial and national PROMs strategies and support the use of PROMs in the Canadian healthcare system [15, 16].

### PROMs in clinical practice

Although PROMs have become widely used in clinical effectiveness research, their usefulness in clinical settings is still unclear. In the clinical context, ideally PROMs results would be fed back to clinicians who would use that information to improve patient care, much like routine clinical laboratory measures [17]. However, since PROMs were generally not developed or applied for this purpose, little is known about how they perform in this context. A limited number of randomized controlled trials have examined the impact of using PROMs in clinical practice, particularly in oncology, and found that the routine measurement of PROMs, followed by reporting of results to clinicians and patients, had a positive impact on patient-provider communication, patient satisfaction with care, and HRQL [9, 18–24]. Similarly, in adolescents with type 1 diabetes, the periodic monitoring and discussion of PROMs led to improvement in psychosocial wellbeing, however, this benefit was not sustained after the completion of the intervention [25, 26]. In patients with gastrointestinal disorders, the one-time use of a disease-specific PROM had no impact on patient satisfaction, provider interpersonal skills, or shared decision-making [24].

Systematic reviews of these clinical trials and others suggest that feedback of PROMs to clinicians have an impact on processes of care, may enhance patient-provider relationship, improve communication, and support shared decision-making [27–30]; however, their impact on health outcomes is less apparent [27, 31–33]. Aside from these limited controlled studies examining the clinical effectiveness of PROMs themselves as an intervention, few studies have examined their value and cost-effectiveness to justify their use in clinical practice [34].

Additionally, qualitative studies that explored practical issues surrounding the implementation of PROMs in clinical practice found that demonstrating clinical utility to clinicians is a key factor for successful and effective implementation of PROMs [35, 36]. Other factors identified include the use of technology, having the appropriate infrastructure in place, embedding PROMs into routine workflow, improving the interpretability of PROMs data, and engaging clinicians in the planning phase of the intervention [35]. Finally, a key element identified by clinicians is the linkage of PROMs with tools to support the clinical management of symptoms or problems identified by assessments [37]. For example, if a patient identifies problems with anxiety or depression, clinicians should have readily available treatment protocols to follow to alleviate those symptoms [35, 36]. Although the current evidence on the impact of PROMs on patient outcomes is relatively limited and has several methodological limitations, it generally suggests that the effectiveness of PROMs depends on the function of the PROM and the type of feedback provided.

PROMs can be generic or disease-specific, and each has several advantages and disadvantages [38]. Generic measures are used to compare outcomes across different populations and interventions, particularly for cost-effectiveness studies and health system decision-makers. Disease-specific measures assess the health status of a specific patient population and may be more sensitive for the detection and quantification of changes that are important to clinicians or patients. A common recommendation in clinical research with PROMs is to combine both a generic and a disease-specific measure for use in a particular patient population, especially when PROMs are used to monitor health and how it changes over time. The presumed benefit of this combination is to broaden the scope of measurement and allow for different uses of the measures (e.g., comparison within versus across disease populations). However, evidence to support this recommendation is limited. In fact, in a study examining the usefulness of generic and disease-specific measures of health status in patients with Alzheimer’s, the EQ-5D (a generic measure) was found to be a suitable alternative to a disease-specific measure of health [39]. Another study reported that generic and disease-specific measures performed similarly in assessing changes in HRQL in patients with kidney failure requiring hemodialysis [40]. There is usually some level of overlap between generic and disease-specific measures, and the need for combining both types of measures depends on the target patient population and the constructs assessed by each measure. Given the workload associated with collecting and using PROM data in clinical settings, it is imperative to explore whether one single PROM is sufficient to improve patient-provider communication, processes of care, and ultimately patient outcomes, before recommending the use of a combination of generic and disease-specific measures.

### Justification for PROMs in dialysis care

One of the key areas where PROMs have been implemented is in patients with kidney failure requiring dialysis. The prevalence of chronic kidney disease (CKD) is relatively high in Canada (12.5% or 3 million Canadian adults) and its advanced stages can lead to kidney failure [41]. Kidney failure requiring dialysis is expensive and is associated with poor health outcomes and HRQL. To report symptom burden and HRQL alongside clinical and laboratory measures, it is imperative to measure and report what is important to patients. PROMs could be used to evaluate and monitor patients’ health, inform care planning, and facilitate the introduction of treatments for patients with kidney failure requiring dialysis [42].

Moreover, the implementation of PROMs assessments and integration into the workflow of a busy clinical setting such as dialysis clinics requires a feasible system to capture and report PROMs. This would be facilitated by the development of PROM report cards to present results to clinicians and patients in a manner that is easily understood and interpretable, analogous to clinical laboratory measures used in the hemodialysis setting. We believe that routine hemodialysis care provides the ideal setting for implementation and rigorous evaluation of a PROM intervention, and as such, we are conducting a pragmatic, cluster randomized controlled trial (RCT) of a PROMs intervention. Our comprehensive evaluation will assess the effectiveness of this intervention, the adoption and implementation in the busy clinical setting of hemodialysis units, as well as the cost-effectiveness of PROMs in routine hemodialysis care.

## Aims of the research

The overall aim of this study is to explore the usefulness of integrating PROM assessments in the clinical management of hemodialysis patients. Specifically, the primary objective is to determine the effects of routine measurement and reporting to clinicians of PROMs, namely, the Edmonton Symptom Assessment System- revised: Renal (ESAS-r: Renal) [43] [44], or the Integrated Palliative care Outcome Scale – Renal (IPOS-Renal) [45] and/or the EQ-5D-5L [46] on patient-reported experience, particularly patient-clinician communication, measured by the Communication Assessment Tool (CAT), for patients with kidney failure requiring hemodialysis. The secondary objectives are as follows:

i. To determine if there is a difference in patient-reported experience and other outcomes induced by a condition specific (ESAS-r: Renal /IPOS-Renal) versus a generic (EQ-5D-5L) PROM;
ii. To determine the effects of routinely measuring and reporting the ESAS-r: Renal/IPOS-Renal and/or the EQ-5D-5L on the clinical management of symptoms;
iii. To determine the effects of routinely measuring and reporting the ESAS-r: Renal /IPOS-Renal and/or the EQ-5D-5L on overall HRQL;
iv. To determine the effects of routinely measuring and reporting the ESAS-r: Renal/IPOS-Renal and/or the EQ-5D-5L on symptom burden, mental health outcomes and healthcare utilization;
v. To determine the cost effectiveness of routinely measuring the ESAS-r: Renal/IPOS-Renal and/or the EQ-5D-5L for patients with kidney failure requiring hemodialysis;
vi. To explore the perspectives and experience of patients and clinicians with routine measurement and reporting of PROMs in clinical practice.

## Methods

### Study design

**Fig. 1:**
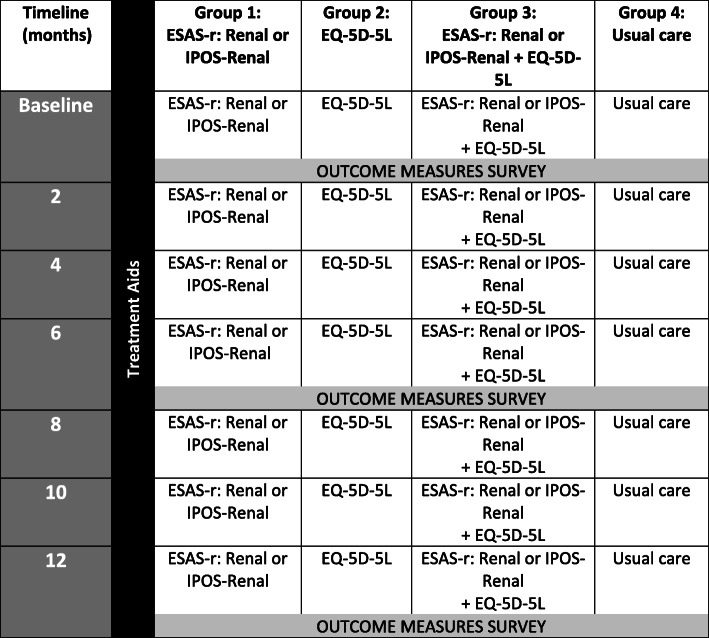
**Schematic of overall design for the EMPATHY Study.** *Outcome measures survey includes: Communication Assessment Tool (CAT), Patient Assessment of Chronic Illness Care 11-items questionnaire (PACIC-11), Patient Health Questionnaire 2-item (PHQ-2), General Anxiety Disorder 2-items questionnaire (GAD-2), Edmonton Symptom Assessment System – revised: Renal (ESAS-r: Renal) or Integrated Palliative care Outcome Scale – Renal (IPOS-Renal), and/or EQ-5D-5L

To date, the effect of PROMs on patient outcomes were generally assessed in conventional parallel-group RCTs of individuals. Few convincingly demonstrated benefits to patient-important outcomes, which might be in part due to contamination effects and small sample sizes [47]. This study uses a cluster RCT design, with clustering occurring at the level of individual hemodialysis units, to reduce potential contamination between the study groups (Fig. 1). EMPATHY is an open-label RCT, since blinding is not feasible [48]. Consequently, research staff, clinicians, and all patients are aware of group allocation. Dialysis units, stratified by three regions (Northern Alberta, Southern Alberta, and Ontario), were randomly allocated using random number sequences with all units from a region randomized on the same day. Dialysis units were allocated in a 1:1:1:1 ratio into four groups:

- Group 1: Patients complete ESAS-r: Renal or IPOS-Renal
- Group 2: Patients complete EQ-5D-5L
- Group 3: Patients complete both ESAS-r: Renal or IPOS-Renal and EQ-5D-5L
- Group 4: Usual care (i.e. the control group). To assess study outcomes, patients complete the PROMs at baseline, 6 and 12 months, but these data are not reported back to clinicians or patients.

### Interventions

**Fig. 2:**
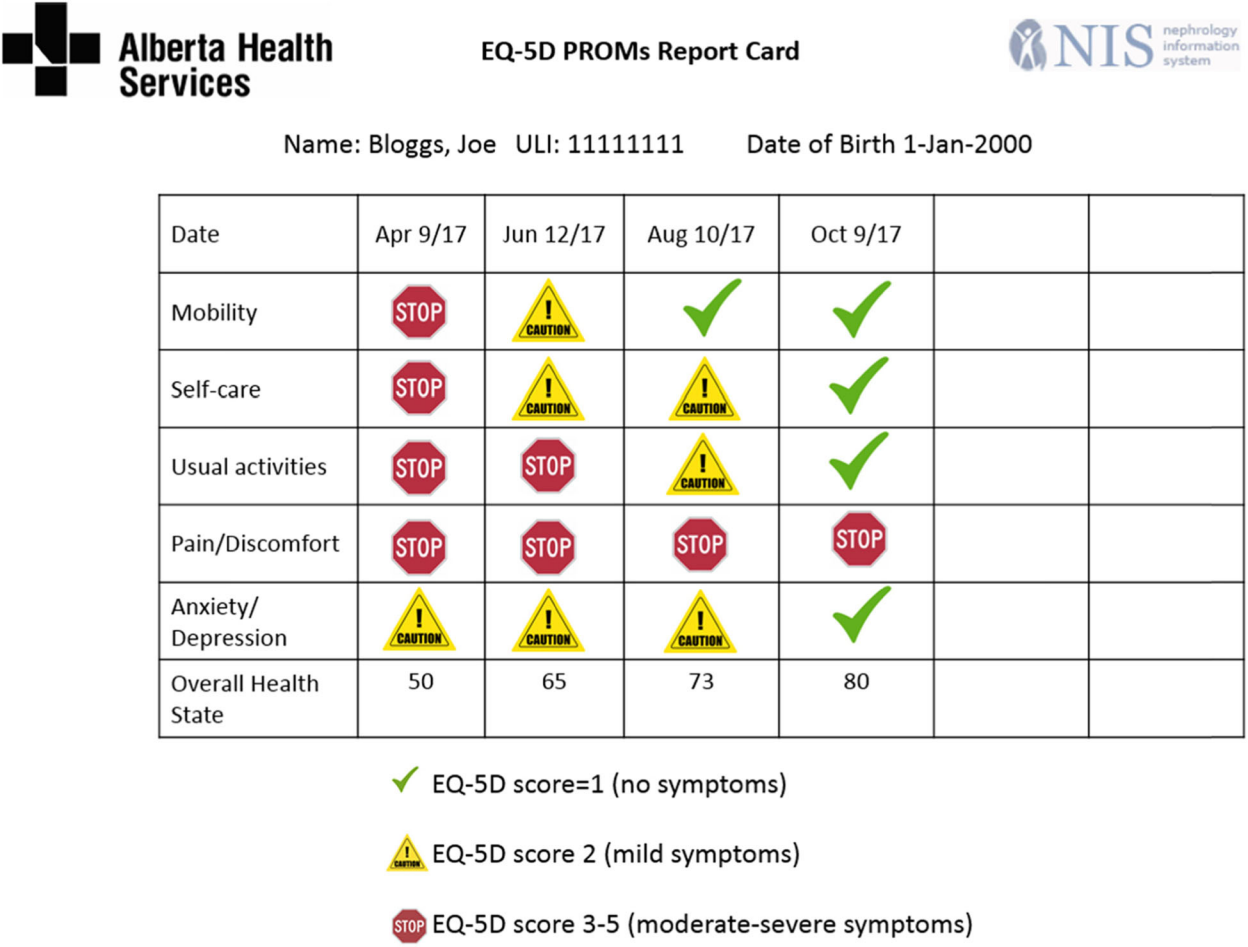
Sample EQ-5D-5 L PROMs Report Card

Groups 1–3 will collect their allocated PROM(s) from all eligible patients every 2 months, for 12 months (a total of 7 assessments). PROMs are administered by pen and paper or through an electronic platform on a tablet in accordance with hemodialysis unit resources. In Alberta, the electronic medical records (EMRs) generate a PROM report card with a symbol scheme for nurses to follow up with clinical management where applicable. The symbol scheme comprises of a check mark for no problems/symptoms (ESAS-r: Renal = 0, IPOS-Renal = 0, EQ-5D-5L = 1) a caution sign for mild problems/symptoms (ESAS-r: Renal = 1–3, IPOS-Renal = 1, EQ-5D-5L = 2) and a stop sign for moderate-severe symptoms/problems (ESAS-r: Renal = 4–10, IPOS-Renal = 2–4, EQ-5D-5L = 3–5). The PROM report card is printed and added to the patient’s medical chart for review by clinicians (i.e., nurses, nephrologists, and allied health professionals). The report displays each patient’s most recent PROM scores in comparison with their previous scores, much like lab test results. A sample PROM report card is shown in Fig. 2. In Ontario, EMRs do not have report card capabilities and units store the paper-completed PROM questionnaires in the patient’s medical chart, along with previously completed questionnaires for review by clinicians and the patient. In all locations, PROMs are accompanied by treatment aids for some symptoms assessed (pain, pruritus, restless legs, sleeping problems, tiredness, nausea, shortness of breath, anxiety, and depression). Patients are also offered a copy of their PROM report card (if possible) and patient-facing self-management guides for symptom management. This trial is intended to generate evidence on the implementation within the health systems. While we will aim to maintain the study design and key elements of the intervention as consistently as possible, we will allow for the interventions to be tailored to local health systems, accommodating considerations such as the clinical work flow, information systems and capabilities, and internet/WiFi access (Table 1).

**Table 1:** Intervention similarities and differences between renal programs

**Setting**	•All three renal programs will implement the intervention in in-centre hemodialysis units only, excluding home hemodialysis and peritoneal dialysis units •The three geographically based renal programs have similar models of multi-disciplinary care
**Disease-specific PROM Use**	•AKC-N and ORN will use the ESAS-r: Renal •ACK-S will use the IPOS-Renal
**Generic PROM Use**	All renal programs will use the EQ-5D-5 L
**Frequency of PROMs**	All renal programs will administer the PROM(s) every 2 months
**Mode of PROMs Administration**	•ORN: clinicians will administer PROMs by paper and store in patients’ charts •AKC-N: clinicians will administer PROMs by paper and then enter PROMs results into their EMR •AKC-S: clinicians will administer PROMs by iPad, directly entering results into their EMR
**PROMs Report Cards**	•AKC-N and AKC-S will generate a PROMs report card with a symbol scheme categorizing symptoms/problems into ‘no symptom/problem present’, ‘mild symptom/problem’ and ‘moderate-severe symptom/problem’ •ORN will simply use the completed PROM(s) for review and discussion
**Treatment Aids**	Each renal program developed their own symptom guidelines for clinicians and patient information handouts
**Clinical Workflow**	All renal programs will deliver the EMPATHY intervention in 3 phases: 1. Screening: PROM(s) are administered every 2 months 2. Assessment: Clinician reviews PROM(s) results with the patient and discuss where management is needed 3. Management: Clinician uses the treatment aids to manage symptoms, as applicable
**Outcome Measures Survey Frequency**	All renal programs will collect the outcome measures through a survey collected at baseline, 6 months, and 12 months
**Outcome Measures Survey Mode of Administration**	•AKC-N and AKC-S will administer the outcome measures survey by iPad, with a paper back-up (e.g., where wifi access not available) •ORN will administer the outcome measures survey by paper
**Clinician Training**	•Each renal program will deliver their own training of the intervention to clinicians as the intervention is slightly different across the renal programs •The overall delivery of education between the three programs will be very similar, using a ‘train-the-trainer’ approach, whereby clinical leaders will first be trained at each site and these leaders will then train relevant clinicians using their usual mechanisms of disseminating new clinical information/policy and procedures •Within each renal program, each unit will name a staff member to act as the ‘EMPATHY site lead’ to champion the trial in the unit and act as a liaison between the unit and the research team

### Hypotheses

We hypothesize that the measurement and reporting of the ESAS-r: Renal or IPOS-Renal, EQ-5D-5L or a combination every 2 months will lead to improved patient-clinician communication in dialysis units. We also hypothesize that this will lead to reduced symptoms and improved mental health and HRQL. Furthermore, we anticipate that this intervention will be cost-effective and will be acceptable and feasible in the hemodialysis units’ environment.

### Setting and population

Dialysis programs frequently provide administrative functions for separate hemodialysis units; however, the unit of randomization is the dialysis unit, not dialysis programs or individual patients. In total, 44 units from three renal programs were randomly allocated to one of the four study groups. We enrolled all eligible hemodialysis units in Alberta, including 17 hemodialysis units from the Alberta Kidney Care - North (AKC-N) program and 12 units from the Alberta Kidney Care - South (AKC-S) program. Of Ontario’s 92 hemodialysis units, 15 enrolled in the study, based on voluntary acceptance of invitation to participate.

### Dialysis unit eligibility criteria

Hemodialysis units meeting the following inclusion criteria were included in this study:

- The unit provides chronic in-center hemodialysis;
- Hemodialysis clinicians are willing to review individual patients’ routinely collected ESAS-r: Renal or IPOS-Renal and/or EQ-5D-5L as part of routine patient assessment;

Hemodialysis units are excluded if they meet any of the following criteria:

- The unit is unable to administer the ESAS-r: Renal or IPOS-Renal and EQ-5D-5L to in-centre hemodialysis patients as part of clinical workflow to all possible patients;
- The unit is part of a long-term care facility
- There are 5 or fewer patients treated at that unit
- Lack of WiFi connectivity (Alberta only)

### Patient eligibility criteria

All patients undergoing chronic hemodialysis who are 18 years or older at the start of the study and are willing and able to complete the PROMs as part of the trial are eligible to participate in this study. Patients with cognitive impairment, undergoing acute dialysis, or transiently dialyzing in the unit are not included. Simplified Chinese, Punjabi, and Vietnamese PROMs and study surveys were provided. Patients that had other language barriers had family help them complete their PROMs assessments and/or study surveys or the health system interpretive services were used. For patients with eyesight issues, their family members or nurses helped them complete the PROMs and study surveys.

### Description of PROMs

#### Disease-specific PROMs

*ESAS-r: Renal:* The Edmonton Symptom Assessment System, or ESAS, is a clinically validated and reliable symptom measurement tool, extensively used for a number of chronic diseases including cancer, heart disease, and kidney disease (Table 2). The original tool was developed by the Regional Palliative Care Program, Capital Health in Edmonton, Alberta [49]. The ESAS-r: Renal was modified from the original ESAS and validated for use with CKD patients in Canada to assess symptom burden [44]. Despite its widespread use in dialysis units in Canada, the use of ESAS-r: Renal has largely been limited to measurement of symptoms without systematic reporting of results to clinicians.

**Table 2:** Comparison of PROMs in the EMPATHY Trial

	ESAS-r: Renal	IPOS-Renal	EQ-5D-5 L
**Type of PROM**	Disease-specific (renal) measure	Disease-specific (renal) measure	Generic, preference-based HRQL measure
**Use by Renal Programs**	•AKC-N •ORN •Study groups 1 and 3	•AKC-S •Study groups 1 and 3	•AKC-N •AKC-S •ORN •Study groups 2 and 3
**Measurement Properties**	•Clinically validated and reliable •Sensitive to change	•Clinically validated and reliable •Sensitive to change	Limited evidence for clinical use •Produces utility scores •Comparison with any population, including population norms
**Content**	Symptoms: •Pain •Tiredness •Drowsiness •Nausea •Lack of appetite •Shortness of breath •Depression •Anxiety •Wellbeing •Itch •Problems sleeping •Restless legs	Symptoms: •Pain •Shortness of breath •Weakness or lack of energy •Nausea •Vomiting •Poor appetite •Constipation •Sore or dry mouth •Drowsiness •Poor mobility •Itch •Difficulty sleeping •Restless legs •Changes in skin •Diarrhea •Anxiety •Depression Other: Information needs, practical concerns, family anxieties, and overall feeling of being at peace	Problems: •Mobility •Selfcare •Usual activities •Pain/discomfort •Anxiety/depression Visual Analogue Scale: overall health state
**Number of items/dimensions**	12	26	5 + VAS
**Scale**	0–10	0–4	5 dimensions: 1–5 categorical Index score: 0-dead, 1-full health VAS: 0–100

#### IPOS-renal

The Palliative care Outcome Scale, or POS, was developed in 1999 [50] for use with patients with advanced disease, and to improve outcome measurement by evaluating important outcomes in palliative care (Table 2). The Integrated Palliative care Outcome Scale, or IPOS, integrates questions from three different POS measures, incorporating symptoms, information needs, practical concerns, family anxieties, and overall feeling of being at peace. The IPOS-Renal contains all the elements of the IPOS, and the most common symptoms renal patients experience. The IPOS-Renal has also demonstrated validity and reliability and has been studied in dialysis and non-dialysis CKD patients in Australia [45].

### Generic PROM: EQ-5D-5L

The EQ-5D-5L is a generic preference-based measure of HRQL and has been selected as the PROM of choice in many clinical areas and settings (Table 2). The EQ-5D-5L includes a health status classification system with five dimensions (mobility, self-care, usual activities, pain/discomfort, anxiety/depression), each with five levels of problems (1 = none, 2 = mild, 3 = moderate, 4 = severe, 5 = extreme), describing 3125 distinct health states [46] [51]. An index score for each health state can be calculated using population preferences [52], which can then be used to estimate quality-adjusted life years (QALYs) in economic evaluations of health interventions or innovations [53]. Empirical evidence on the clinical application of the EQ-5D-5L with regards to individual patient care is limited, but a recent review of PROMs in CKD recommended the EQ-5D-5L for use in this patient population [54].

### Treatment aids

Treatment aids are assessment and treatment resources developed by expert clinicians for the management of certain symptoms and disorders. Each renal program developed treatment aids specific to symptoms assessed by the ESAS-r: Renal or IPOS-Renal (e.g., itchiness, restless leg syndrome, nausea) and the EQ-5D-5L (e.g., pain, anxiety/depression). Treatment aids are intended to support clinicians in the assessment and management of symptoms identified by these PROMs and will be made available for all clinicians at all study sites regardless of the group to which they are randomized.

Patient-facing materials are also provided to patients, to help them better understand the reasons why their symptom/problem may have developed, treatment options that might be considered, as well as self-care activities they can undertake to alleviate the symptom or problem.

### Clinician training

**Fig. 3:**
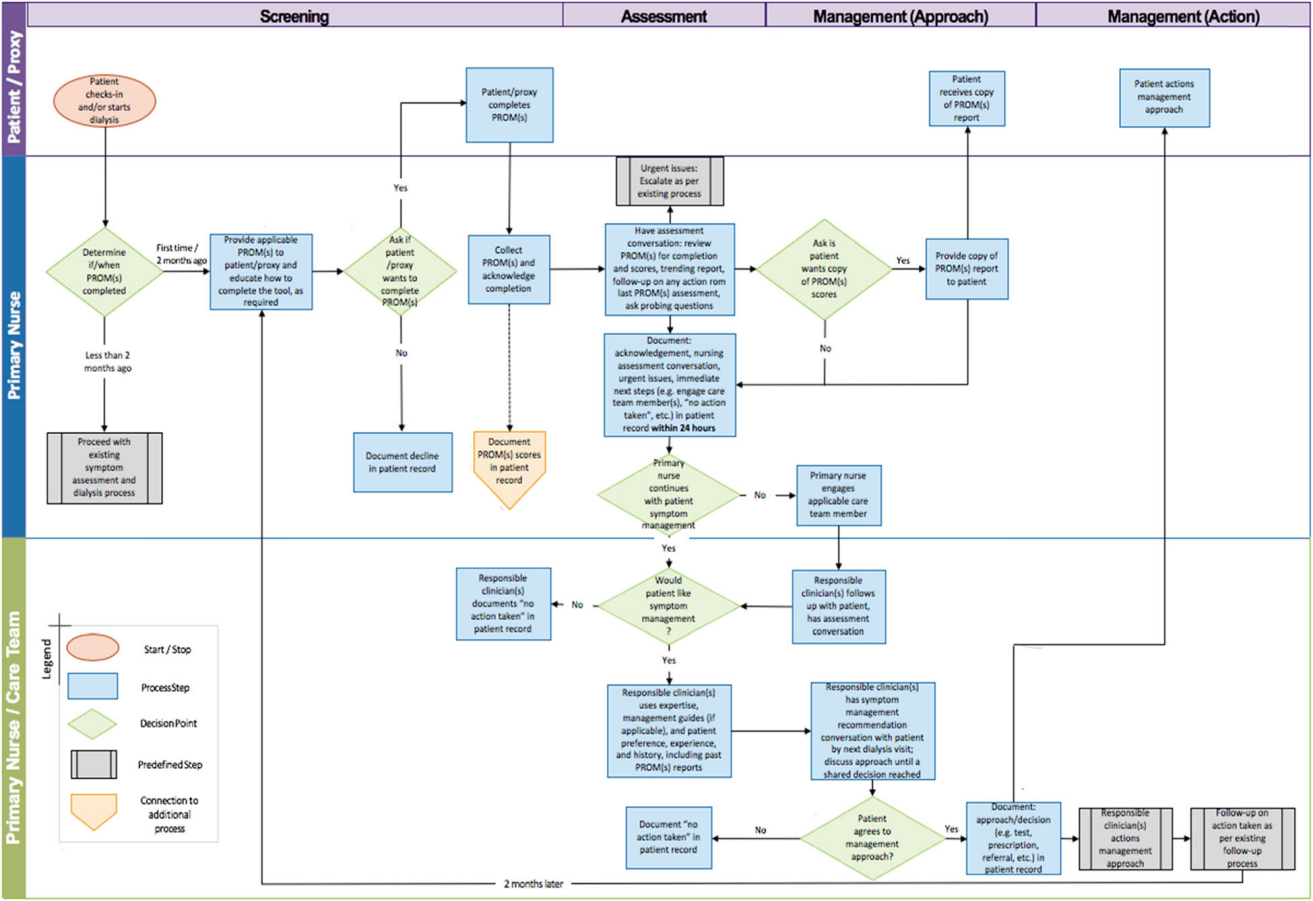
Nurse Workflow of EMPATHY Intervention

Nurses were responsible for administering PROMs to patients. They received training on the use of the ESAS-r: Renal or IPOS-Renal and/or EQ-5D-5L, depending on their study group allocation, which included an overview of the instruments, interpretation of patients’ scores from one measurement and changes in scores over time, the use of PROMs data during clinical visits (i.e. referring to treatment aids or clinical guidelines), as well as data entry in the EMR, if applicable. A toolkit for using PROMs in the dialysis unit was developed and shared with all clinicians in all participating dialysis units. A train-the-trainer model was adopted, where clinical nurse educators will receive training and subsequently provide on-site training to other nurses. Training was administered in a series of webinars and workshops. Figure 3 demonstrates the workflow whereby nurses were trained. Nephrologists received training through in-person presentations and print materials.

### Primary and secondary outcome measures

The *primary outcome* in this study is patient-provider communication, which is assessed using a modified version of the Communication Assessment Tool (CAT) [55].

*Secondary outcomes* include:

- HRQL, assessed using the EQ-5D-5L. [46].
- Symptom burden, assessed using the ESAS-r: Renal [44] or IPOS-Renal [45] and symptom management through the EMR.
- Anxiety and depressive symptoms, assessed using the Generalized Anxiety Disorder 2-items questionnaire (GAD-2) [56] and the Patient Health Questionnaire 2-items (PHQ-2) [57], respectively.
- Patient experience with their disease management, assessed using the Patient Assessment of Chronic Illness Care 11-items questionnaire (PACIC-11) [58].
- Healthcare utilization (e.g., physician, emergency department and hospitalization), assessed through linkage with provincial administrative health care databases at the individual patient-level.

Demographic characteristics and the outcome measures are collected at baseline, 6-months, and 12-months. Data on medical history, dialysis history, and clinical measures are collected from patient charts.

### Sample size and power

The EMPATHY trial is a pragmatic clinical trial with a sample size determined based on eligibility of hemodialysis units and its encompassing patients. Our intention was to enroll all eligible dialysis units within the province of Alberta, and a sample of eligible units in Ontario. Therefore, we did not calculate an optimal sample size for adequate power to detect statistical significance. From 44 dialysis units, we will be utilizing data from approximately 3000 patients. Based on an estimated intraclass correlation of 0.1 and a standard deviation for the CAT (primary outcome measure) of 0.74 (based on pilot data from HD patients in Alberta), this will provide 80% power to detect an effect size of 0.23 with a two-tailed test at typical level of significance, α = 0.05 [59]. While this is a small effect size for the primary outcome, we are nonetheless confident in the design, given that this is a pragmatic quality improvement study, involving 44 dialysis units from three renal programs with several secondary objectives related to patient experience and outcomes.

### Quantitative trial data analysis

The primary analysis will assess change in the overall CAT score, between study groups from baseline to 12 months using a linear mixed effects model, with the mean CAT for the dialysis unit at baseline as a fixed effect covariate, and dialysis unit (cluster) as a random intercept. Ideally, analysis should use individual patients’ baseline CAT and follow-up CAT rather than the unit mean baseline CAT to compute change over time. However, due to concerns by the regional renal programs to maintain anonymity of experience data as well as the natural drop-in and drop-out of patients in the dialysis units due to patient deaths and initiation of new patients onto dialysis, individual CAT surveys were made completely anonymous. As such, our primary analytic plan will be based on changes in mean CAT score at the unit level, from baseline to 12 months. The primary analysis will be based on all of the PROMs intervention groups combined compared to the control (no PROMs intervention) group as fixed effects. Any units that drop-out after randomization but prior to initial data collection will be excluded. We will employ last observation carried forward methods to impute missing data where appropriate. As one of our secondary objectives, assessing differences between specific and generic PROMs, we will compare the individual interventions groups (i.e., ESAS-r: Renal /IPOS alone, EQ-5D-5D alone, or the combination) to the control group.

As part of our secondary analyses, we will also compare the proportion of patients reporting symptoms/problems on the ESAS-r: Renal or IPOS-Renal and EQ-5D-5L and the management of such symptoms/problems. Through clinical records, we will link PROMs scores with the use of specific symptomatic treatments as per the treatment aids. We will obtain medication lists, allied health referrals, and chart notes to capture how symptoms are being treated. Because the PROMs data will be collected as part of the clinical record, and linked with individual patient-level data, we will be able to analyze these data using logistic mixed-effects regression models adjusting for individual patient-level covariates. Each renal program (i.e., AKC-N, AKC-S and ORN) will be analyzed separately then we will use meta-analyses to analyze the data from each renal program.

### Economic evaluation

A cost-effectiveness analysis will be employed of the different study groups, compared to the usual care group as the reference case. The incremental costs and incremental QALYs of the different interventions relative to the usual care group will be compared to calculate incremental cost per QALYs gained ratios [60, 61]. Costs will be estimated for patients in the different study groups, including treatments received by patients, taking the perspective of the provincial health care system, personnel costs for training and delivery of the PROMs assessments, and subsequent health care utilization for patients in each of the study groups. Health care utilization will be estimated through linkage with provincial administrative health care databases at the individual patient-level. Costs associated with provision of the intervention, including training and nursing time, will be estimated through observations for time and valued using standard staffing wage scales for the respective geographic regions. QALYs will be calculated based on changes in the EQ-5D-5L index score, using the Canadian preference weighting [62]. Discounting or inflationary factors will not be applied as the analysis will be limited to the period of the study itself (i.e., 12 months). Self-reported survey and clinical data will be linked to administrative data on healthcare utilization (e.g., number of hospitalizations, number of emergency department visits, number of family physician and specialist visits, cost), one-year prior and one-year after the intervention starts. Linkage to lab and medication datasets will also be performed where available.

### Qualitative sub-studies

Separate qualitative assessments will be undertaken within the regional dialysis programs. Within AKC-N, we will employ an interpretative description approach [63], ascertaining the experiences of patients and clinicians that will participate in the trial, in the routine measurement and reporting of PROMs and the use of the treatment aids, and in relation to communication and clinical management. Interpretative description is an inductive approach used to understand clinical phenomenon with the end goal of informing practice. Of particular interest will be an exploration of how the PROMs intervention is implemented within the workflow of the different clinical settings amongst the dialysis units. This qualitative study will enhance the larger trial by examining patient and clinician experiences regarding the study’s primary outcome of patient-provider communication. A variety of data collection methods will be employed, including clinic observations, interviews, and open-ended comments from patients on the study survey. These data will be managed using Atlas.ti and thematically analyzed.

A distinct qualitative study will also be undertaken within AKC-S. Using a qualitative descriptive approach [64, 65], we will explore the PROM implementation process among hemodialysis units randomized to an intervention group of the trial. In this study, our aim is to characterize perceived barriers and facilitators to effective and sustainable use of the PROM intervention in routine hemodialysis care. Interviews and focus groups with hemodialysis patients and clinicians will be guided by the Consolidated Framework for Implementation Research (CFIR), whose domains (i.e., intervention characteristics, outer/inner setting, individual characteristics, and implementation process) can be used to inform and/or evaluate implementation strategies [66]. Directed observations in hemodialysis units of PROMs assessments and feedback will provide additional contextual information about the setting, workflow, and PROMs integration in care. Data will be managed using NVivo 11 and thematically analyzed [67].

## Patient engagement

Patient engagement in this study was guided by Can-SOLVE CKD Network strategies and guidelines. Development of the research question, outcome measures, and study design were informed by a series of meetings held with the EMPATHY research team, which included people with lived experience of kidney failure on hemodialysis. These patient partners helped develop and review all the symptom information handouts for patients. They also evaluated the overall burden of study participation during the design process and helped determine the type and frequency of PROM administration. Additionally, patient partners prioritized focusing on mental health in our intervention and our study outcomes. Mental health treatment aids were developed and mental health outcome measures were added to the study survey as a result. The scope of this study was significantly impacted by the addition of patient partners on the study team.

## Study timelines

The three renal programs implemented EMPATHY on different timelines. AKC-N implemented EMPATHY in September 2018 with all units starting at once. AKC-N completed the 1-year trial period in October 2019. AKC-S and ORN implemented EMPATHY with a phased approach, meaning groups of units started the trial at different points. AKC-S started phasing units into the trial in January 2019 and ORN in April 2019. ORN and AKC-S will complete the trial by the end of 2020.

## Data storage and security

Any hard copies of PROMs reports are kept in patients’ charts. Study data is stored in the EMR (Alberta) or an Excel file (Ontario) and transferred to the research team using secure mechanisms, with appropriate encryption. Electronic data is password protected, saved on a secure university server and accessed only by members of the research team. All research team members follow the relevant codes of practice concerning confidentiality, information security management and records management.

Outcome measure data was collected by paper surveys or by iPads and directly entered into REDCap, a web-based application for electronic data collection. The REDCap database is saved on a secure Alberta Health Services (health authority) server. Using REDCap limits the amount of paper-based data, further ensuring data integrity and safety. Paper surveys are sent to the research team by courier mail, fax with cover-page, encrypted email, or in-person and are manually entered into the REDCap (Alberta) or Excel (Ontario) database by a member of the research team. A 25-year data retention policy will be adopted for hard-copy data and electronic records, as per Health Canada regulations.

The present protocol fulfilled the Standard Protocol Items: Recommendations for Intervention Trials (SPIRIT) checklist.

## Discussion

The evaluation of PROM interventions such as those described in this protocol can be challenging given the nature of clinical practice in busy hemodialysis units, the variations in organization and clinical workflow across units, as well as regional programs. We will study the implementation of PROM interventions in real-world health settings. Thus, we will adopt a pragmatic approach and accept the necessary variations in implementation, while attempting to retain as many of the core elements of the interventions across the programs. Furthermore, given the desire to maintain anonymity of patient-reported experience, our primary analytic strategy will be limited to comparison of the outcome measures at the group level. These pragmatic approaches to the design of this implementation study limit the rigour of the evaluation of the efficacy of PROM interventions, but on the other hand, enhance our evaluation of real-world effectiveness. Along with the planned qualitative assessments, the information gained from this implementation trial will provide valuable information for the regional dialysis programs in enhancing and sustaining the PROM interventions in the most appropriate manner.

A question that is often raised with the implementation of PROM programs is the choice of the measures. We are choosing to implement a combination of specific and generic measures. While a secondary objective is to compare these types of measures, it is possible that the differences (e.g., breadth or scope) between measures matter less than either approach simply serving as a mechanism for engaging patients and clinicians in a more focused discussion on symptoms or problems. This is the intention with our primary analytic strategy of combining the intervention groups to compare against the control group with no PROM intervention. There may be other reasons, beyond the patient-clinician interaction, however, that guide decisions on the choice of measures. For example, programs may be interested in evaluating the overall quality and efficiency of services, and may therefore be interested in a measure that provide data to support economic evaluation. In this case a generic preference-based measure such as the EQ-5D provides an overall index score compatible with that purpose, which may not be available with measures assessing symptom burden.

A further question faced by clinical programs is the choice of any particular measure for either generic or specific health status. Indeed, within our trial protocol, we will have two different instruments assessing symptom burden. The ESAS-r: Renal will be used in AKC-North and the ORN, based on previous use of this measure in dialysis and other programs locally and across Canada; the IPOS will be implemented in AKC-South, where there was no previous PROM collection, and as a choice informed by regional patient engagement. Similarly, we chose to use the EQ-5D-5L as a generic measure, which was recommended for this patient population in a review paper that was available at the time we planned our interventions [54]. A more recent review of measurement properties for PROMs for adults with chronic kidney disease recommends the KDQOL-SF or KDQOL-36 [68]. The authors note, however, that the available evidence for the reliability and validity of these measures is limited, calling for further research to close this information gap [68]. Nonetheless, as we noted earlier, it may be that subtle differences between measures matter less than implementing any measure and linking these with actionable clinical pathways to alleviate bothersome symptoms and improve patients’ well-being.

Incorporating PROMs into clinical practice seems like an entirely appropriate strategy to engage patients and enhance their role in decisions regarding their care and outcomes. However, how this is best done is uncertain and requires a substantial allocation of healthcare resources. It is important to rigorously evaluate such interventions and investments to ensure they provide value for patients and health systems. The results of this trial will guide the use of PROMs in Alberta and Ontario dialysis units. These results will also inform the use of PROMs in routine dialysis care nationally and internationally and potentially for the care of other chronic disease patient populations. Dissemination of findings will be undertaken through meetings, reports, academic papers, and conference presentations.

## Data Availability

The data that supports the findings of this study are available from the University of Alberta, University of Calgary and McMaster University but restrictions apply to the availability of these data, which were used under license for the current study, and so are not publicly available. Data are however available from the corresponding author upon reasonable request and with permission of the aforementioned universities.

## Abbreviations

AKC-N: Alberta Kidney Care – North
AKC:S: Alberta Kidney Care – South
CAT: Communication Assessment Tool
CFIR: Consolidated Framework for Implementation Research
CIHI: Canadian Institute for Health Information
CKD: Chronic kidney disease
EMR: Electronic medical record
ESAS-r: Renal: Edmonton Symptom Assessment System-revised: Renal
GAD-2: Generalized Anxiety Disorder 2-item
HRQL: Health-related quality of life
IPOS-Renal: Integrated Palliative care Outcome Scale – Renal
ORN: Ontario Renal Network
PACIC: Patient Assessment of Chronic Illness Care
PHQ-9: Patient Health Questionnaire 9-item
PROMs: Patient-reported outcome measures
QALY: Quality-adjusted life year
RCT: Randomized controlled trial
